# Skin telocytes *versus* fibroblasts: two distinct dermal cell populations

**DOI:** 10.1111/jcmm.12671

**Published:** 2015-09-28

**Authors:** Yuli Kang, Zaihua Zhu, Yonghua Zheng, Weiguo Wan, Catalin G Manole, Qiangqiang Zhang

**Affiliations:** a Department of Dermatology, Huashan Hospital, Fudan UniversityShanghai, China; b Division of Rheumatology, Huashan Hospital, Fudan UniversityShanghai, China; c Department of Respirology, Xinhua Hospital Affiliated to Shanghai Jiao Tong University School of MedicineShanghai, China; d ‘Carol Davila’ University of Medicine and PharmacyBucharest, Romania; e “Victor Babeş” National Institute of PathologyBucharest, Romania

**Keywords:** human dermis, telocytes, telopodes, fibroblasts, cytokines

## Abstract

It is already accepted that telocytes (TCs) represent a new type of interstitial cells in human dermis. In normal skin, TCs have particular spatial relations with different dermal structures such as blood vessels, hair follicles, arrector pili muscles or segments of sebaceous and/or eccrine sweat glands. The distribution and the density of TCs is affected in various skin pathological conditions. Previous studies mentioned the particular (ultra)structure of TCs and also their immunophenotype, miR imprint or proteome, genome or secretome features. As fibroblast is the most common intersitital cell (also in human dermis), a dedicated comparison between human skin TCs and fibroblasts (Fbs) was required to be performed. In this study, using different techniques, we document several points of difference between human dermis TCs and Fbs. By transmission electron microscopy (TEM) and scanning electron microscopy (SEM), we demonstrated TCs with their hallmark cellular prolongations – telopodes. Thus, we showed their ultrastructural distinctiveness from Fbs. By RayBio Human Cytokine Antibody Array V analyses performed on the supernatant from separately cultured TCs and Fbs, we detected the cytokine profile of both cell types, individually. Two of 79 detected cytokines – epithelial-derived neutrophil-activating peptide 78 and granulocyte chemotactic protein-2 – were 1.5 times higher in the supernatant of TCs (comparing with Fbs). On the other hand, 37 cytokines were at least 1.5 higher in Fbs supernatant (comparing with TCs), and among them six cytokines – interleukin 5, monocyte chemotactic protein-3 (MCP-3), MCP-4, macrophage inflammatory protein-3, angiogenin, thrombopoietin – being 9.5 times higher (results also confirmed by ELISA testing). In summary, using different techniques, we showed that human dermal TCs and Fbs are different in terms of ultrastructure and cytokine profile.

## Introduction

Telocytes (TCs) are already acknowledged as new and distinct type of interstitial cells of human dermis 1–3. The presence of TCs was also documented in the stroma of different other organs, hitherto 4–22. Moreover, abundant published data regarding TCs in other organs also indicate multiple points of difference between TCs and the other interstitial cells 23–26. (Ultra)structurally, in dermis (as in other organs), the distinctiveness of TCs is given by their particular cellular prolongations – telopodes (Tps). Telopodes are abruptly emergent very long prolongations of unequal calibre, alternating thin segments (podomers) and dilated segments (podoms) 1. A new and powerful technique – focused ion beam scanning electron microscopy (SEM) – demonstrated some diversity of Tps 3D conformation 2, and also brought additional information on the ultrastructure of TCs. Within human dermis, TCs have particular relations with blood vessels, nerve endings or elements of the pilosebaceous unit. Moreover, TCs are interconnected forming a 3D network either physically (by homocellular and heterocellular junctions) or *via* paracrine interactions (by microvesicles, ectovesicles or shed vesicles) 1,2,20. It was previously hypothesized the functional involvement of TCs in intercellular signalling and communication either in normal skin or in skin under pathological conditions 20. The normal density and distribution of TCs is affected in skin pathological conditions (*e.g*. systemic sclerosis 27,28, psoriasis 29, *etc*.). Furthermore, alterations of normal TCs ultrastructure were described in psoriatic dermis 29.

In connective tissue, fibroblasts (Fbs) have mainly structural functionality, being responsible for production of components of the extracellular matrix: collagen and elastic fibres, ground substance and cytokines 30–32. The ability of protein synthesis is ultrastructurally translated in a euchromatic nucleus and abundance of organelles is involved in production of proteins 29. Fibroblasts are participating in the repair of injured tissues under the influences of fibronectin or Fbs growth factor 33,34.

In this study, we compared TCs and Fbs from human dermis in terms of ultrastructure and cytokine profile. We bring more evidence that in human dermis, TCs and Fbs are two distinct cell population. These data will become a requisite in establishing the details of TCs' functional potential in skin, in health and disease.

## Materials and methods

### Tissue samples

#### For transmission electron microscopy

Biopsies of normal skin were harvested from 10 patients, as previously presented in Manole *et al*. 29.

#### For SEM and cell cultures

Human skin Fbs were purchased from Chinese Academy of Science, Kunming Cell Bank (Cat. no. KCB200537, HSF, Kunming, China). Telocytes cultures were obtained from human dermal samples taken from male patients accepting foreskin resection. The study was approved by the Ethic Committee of Fudan University (Shanghai, China). The informed consents were obtained from all of the patients included.

### Transmission electron microscopy

Small tissue fragments (1 mm^3^) were processed according to a routine Epon embedding procedure as described previously 29. Thin sections (∼60 nm) were examined with a Morgagni 268 transmission microscope (FEI Company, Eindhoven, the Netherlands) at 80 kV. Digital electron micrographs were obtained with a MegaView III CCD and iTEM-SIS software (Olympus, Soft Imaging System GmbH, Münster, Germany). To highlight the TCs and Tps, the TEM image was digitally coloured in blue using Adobe^©^ Photoshop CS3.

### Scanning electron microscopy

The cell samples were fixed in 3% buffered glutaraldehyde for 3 hrs and then washed for three times in PBS (10–15 min. each change). Post-fixation was made in 2% osmium tetroxide for 2 hrs. The dehydration was done using a series of increased concentrations of ethanol. The samples were then transferred to a critical point dryer. The cells were then observed and photographed with a JEOL JSW-6390LV (Tokyo, Japan) scanning electron microscope, at 5 kV.

### Cell culture

Human skin Fbs were cultured in the six-well plate (Corning, Chicago, IL, USA) at a density of 1 × 10^5^/well and maintained at 37°C, in a humidified atmosphere (5% CO_2_ in air). The cells were continuously cultured for 48 hrs in DMEM (Gibco, New York, NY, USA), 2 ml for each well, without foetal bovine serum (FBS; Gibco). For further cytokine antibody array measure, the supernatant was collected and stored at −20°C.

Telocytes isolation and cultures were performed in accordance with the method previously described by Hatta *et al*. 35. The human foreskin tissues samples were harvested under sterile conditions into sterile tubes containing DMEM. The samples were then aseptically rinsed with DMEM and minced into fragments about 1 mm^3^. The fragments were then incubated at 37°C for 4 hrs on an orbital shaker with 1 mg/ml collagenase type II in PBS (Gibco) without Ca^2+^ and Mg^2+^. The reaction of collagenase was terminated by 10% FBS (Gibco). Dispersed cells were separated from non-digested tissue by the filtration through a 40 μm diameter cell strainer (BD Falcon, NJ, USA) and collected by centrifugation at 2000 r.p.m. for 5 min. The cells digested from tissues were plated on 25 cm^2^ plastic culture flasks in DMEM with 10% FBS (Gibco) and 1% penicillin (Sigma Chemical, St. Louis, MO, USA)/streptomycin (Sigma Chemical) at a density of 1 × 10^5^ cells/cm^2^. The cells were maintained at 37°C, in a humidified atmosphere (5% CO_2_ in air) until becoming semi-confluent (usually 4 days after plating). The culture medium was changed every 48 hrs. In the 5th day, the adherent cells were collected and re-plated into a new six-well culture plate (Corning, New York, NY, USA) at a density of 1 × 10^5^/well. The cells were then maintained at 37°C, in a humidified atmosphere (5% CO_2_ in air), and were continuously maintained in DMEM without FBS (Gibco), 2 ml for each well, for 48 hrs. For cytokine antibody array measure, the supernatant was collected and stored at −20°C. Cells were observed and photographed under a light microscope through 40× objective (Olympus 1X51; Olympus, Tokyo, Japan).

### Cytokine antibody array measure

Supernatants, obtained from cultured human dermal TCs and Fbs, were analysed with cytokine antibody array (Human Cytokine Antibody Array V, RayBiotech Inc., Shanghai, China). This study was performed by Kangchen Bio-tech Company (Shanghai, China). Briefly, cytokine array membranes were blocked in blocking buffer for 30 min. and then incubated with samples at room temperature for 1–2 hrs. Samples were then decanted, and membranes were washed. The membranes were incubated with diluted biotin-conjugated antibodies (Cusabio, DE, USA) at room temperature for 1–2 hrs. After the membranes were washed again, 1000-folds diluted horseradish peroxidase-conjugated streptavidin (Proteintech, Chicago, IL, USA) was added and incubation was continued for 2 hrs at room temperature. The membranes were then washed thoroughly and exposed to detection buffer (Raybiotech, Atlanta, GA, USA) in the dark, before being exposed to X-ray film. By comparing the signal intensities, relative expression levels of cytokines could be obtained. The intensities of signals were quantified by densitometry. Biotin-conjugated immunoglobulin G (Cusabio) served as a positive control at six spots to normalize the results from different membranes being compared.

### Data analysis

The SPSS 17.0 software package (SPSS, Chicago, IL, USA) was used to process the data. To calculate a *P*-value for comparisons between two samples, statistical analyses were performed with Student's *t*-test. The threshold of significance was set at *P* < 0.05.

## Results

**Figure 1:**
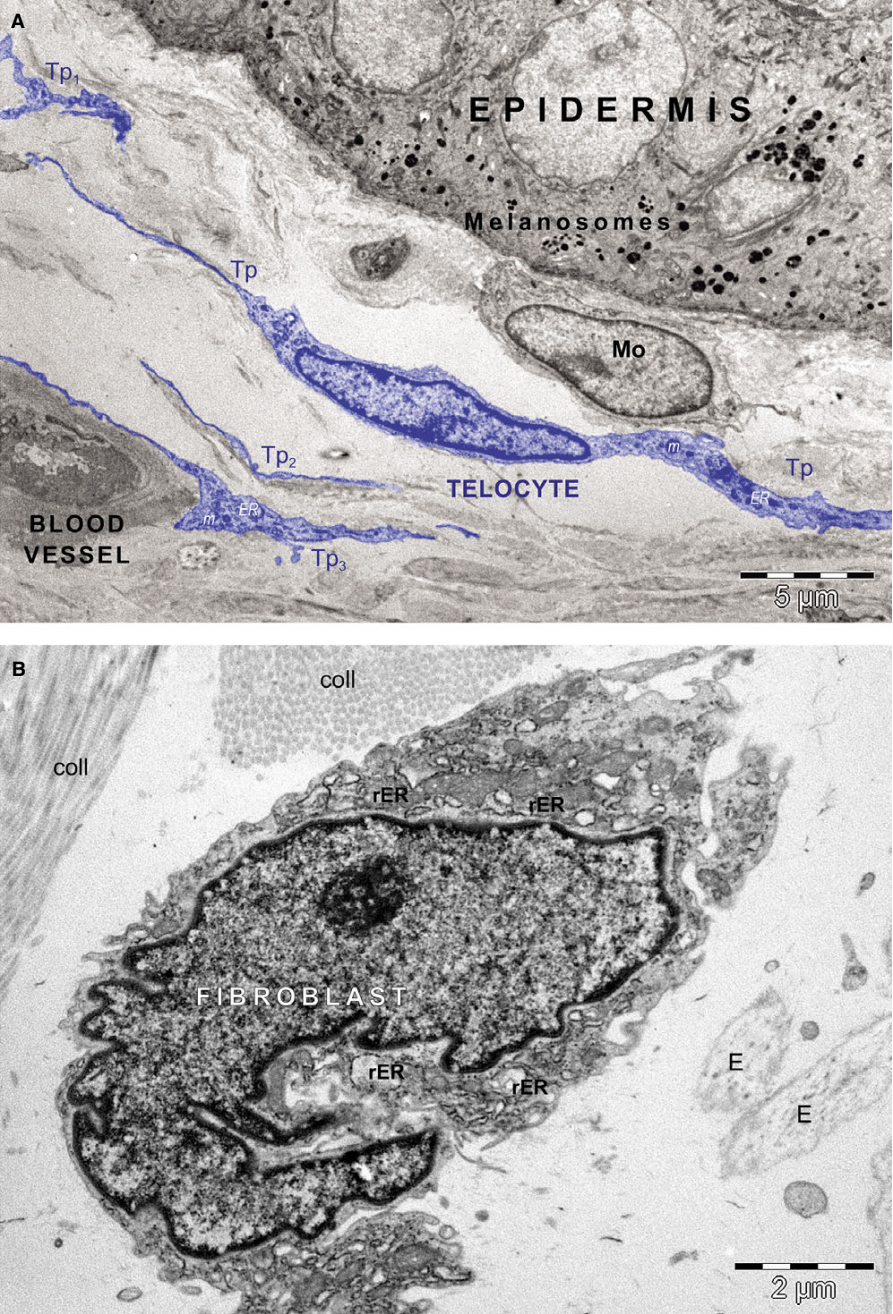
Transmission electron microscopy of the normal human dermis. (A) A telocyte (and its telopodes – Tp) is visible in the papillary dermis, parallel with the basal membrane of the epidermis. Podoms – the dilated segments of Tp – show mitochondria (m) and endoplasmic reticulum (ER) within. A close relation of Tp with a mononuclear cell is visible. However, other TPs – Tp1, Tp2, Tp3 (most probably belonging to other different TCs) run parallel to each other. Moreover, Tp3 is establishing close contacts with the *ab luminal* face of endothelial cells of a capillary. (B) A typical fibroblast is shown in the human papillary dermis. The prominent euchromatic nucleus with visible nucleolus is characteristic. Also, the presence of abundant rough endoplasmic reticulum (rER) is seen. The fibroblast is surrounded by collagen fibres (coll) and elastic fibres (E).

The transmission electron microscopy (TEM) analysis was focused on dermis from healthy donors. Cells with ultrastructure evocative for TCs were recognized in papillary dermis, among other interstitial cells. Commonly, these cells have a small cellular body, containing a large ovoid nucleus rimmed by scarce cytoplasm with rare organelles within, and two visible cellular prolongations (Fig. 1A). The ultrastructural features of these cellular prolongations correspond to that of Tps: characteristically very long and very thin, with string of beads appearance, alternating dilated segments (podoms) and thin segments (podomers). Podoms contain visible mitochondria and endoplasmic reticulum. In papillary dermis, TCs run parallel with the basal membrane of epidermis. Interestingly, TEM analysis confirmed that in papillary dermis, TC was in the close vicinity of mononuclear cells (Fig. 1A). The fine section intercepted other Tps (most probably belonging to other TCs) – Tp_1-3_ – running parallel to each other and all being parallel with epidermis basal membrane. Moreover, Tp_3_ is establishing contacts with the *ab luminal* surface of capillary endothelial cells.

The ultrastructural features of TCs are completely different than that of Fbs (Fig. 1B). In contrast to TCs, the cell body of Fbs is not elongated, being obviously more voluminous and containing a larger amount of cytoplasm in comparison with TCs. Abundant rough endoplasmic reticulum is present within cytoplasm of Fbs. The nucleus of the Fbs is indented and highly euchromatic, containing a visible nucleolus. Fibroblasts are surrounded by bundles of collagen fibrils and elastic fibres.

**Figure 2:**
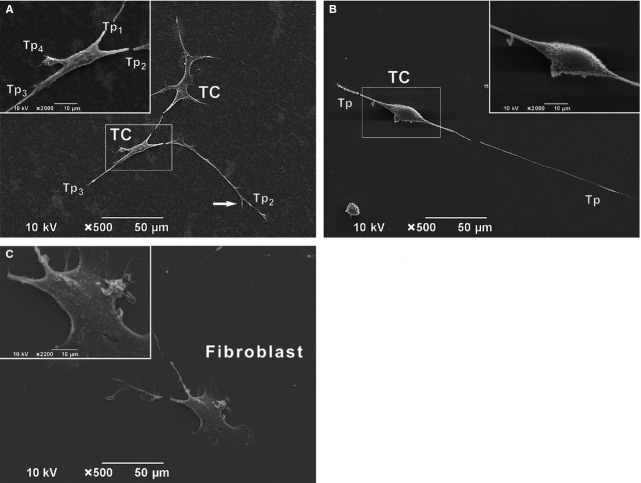
Scanning electron microscopy on cultured cells (A–C). In the primary culture, dermal telocytes (TCs) are featured by a small cell body and very long prolongations. The shape of the cellular body varies from stellate (A) to spindle shape (B) depending on number of Telopodes (Tp). Each Tp emerge suddenly from the cell body and is featured by the considerable length and the moniliform aspect – the alternation of podomers (thin segments) and podoms (dilated segments). For example, in A, the length of the entire visible Tp2 is of about 150 mm and in B, the longest Tps is of about 185 mm. Also, dichotomies are seen along Tps (white arrow) in A. The close vicinity of another TC of similar shape is noted in A. Both TCs seem to establish homocellular junction by their Tps. (C) Adversely, fibroblast is characterized by a larger cellular body with short, cone-like cellular prolongations.

Scanning electron microscopy on primary culture of human dermal TCs and Fbs (days 3–5) shows more evidence on the morphological features of TCs (Fig. 2). The typical morphology is defined by a small cellular body (of various shapes, from stellate shape in Figure 2A to spindle shape in Figure 2B, depending on numbers of Tps) with several (2–4) visible Tps (Tp_1-4_) of variable length (the approximate length of Tp_2_ in Figure 2B is 400 μm). All Tps are of uneven calibre having irregular knob-like dilations – podoms – alternating asymmetrically with thinner segments – podomers. Alongside, Tps present dichotomies of branches (Fig. 2A). TCs, by their Tps, in cell culture also form junctions (Fig. 2A). In contrast, Fbs are characterized by a larger cellular body with two visible short, cone-like prolongations, with large emergence (Fig. 2C).

**Figure 3:**
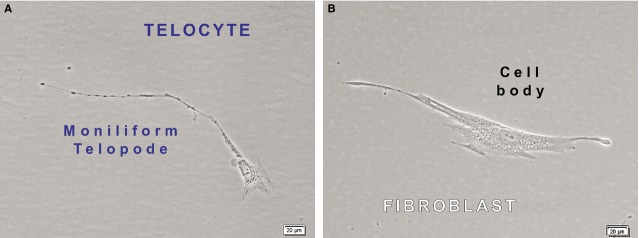
Phase contrast microscopy of cultured cells show the structural differences between dermal telocytes (A) and dermal fibroblasts (B). At the same magnification, the cell body of Telocyte (A) appear smaller than the cellular body of fibroblast (B). In terms of volumes, the nucleus:cytoplasm ratio favours telocytes (A). The slenderness and the characteristic moniliform appearance (alternation of podoms and podomers) of one visible telopode is presented in A. Denser structures (most probably organelles) are present within cytoplasm around nucleus and they are also concentrated at the level of the podoms. The approximate length of the visible telopode is 350 mm. In contrast, the fibroblast has shorter, thicker and cone-like cellular prolongations with no organelles within.

Phase contrast microscopy documented the differences between TCs (Fig. 3A) and Fbs (Fig. 3B). At the same magnification, TCs characteristically present a visible smaller cell body than that of Fbs. The measured length of the Tp in Figure 3A is ∼350 μm, in comparison with the measured cellular prolongation of the Fbs, several tens of μm. Moreover, Tp appears as a very long and slender cellular prolongation of abrupt emergence from the cell body, alternating podoms (dilated segments) and podomers (thin segments), with a string of beads appearance. This is contrasting with the thick and short cone-like silhouette of the Fbs prolongations in Figure 3B.

**Figure 4:**
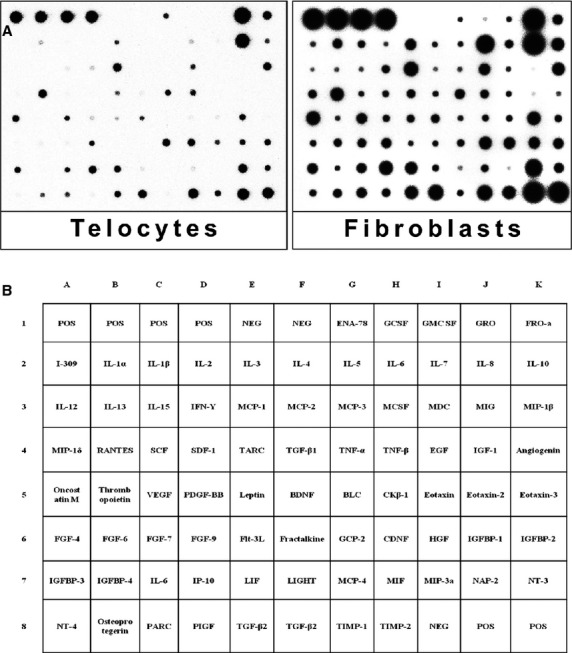
Cytokine profiles of human skin telocytes and fibroblasts. Supernatants of these two kinds of cells were subjected to cytokine antibody array. (A) It presents the results of the array, every black dot represents a cytokine. (B) It is the list of cytokines, each being homologue for every black dot in A. POS: positive; NEG: negative; ENA: epithelial cell-derived neutrophil attractant; GCSF: granulocyte colony-stimulating factor; GM-CSF: granulocyte–macrophage colony stimulating factor; GRO: growth regulated oncogene; I-309: eosinophil chemo attractant protein CCL-1; IFN: interferon; MCP: monocyte chemotactic proteins; MCSF: macrophage colony -stimulating factor; MDC: macrophage-derived chemokine; MIG: monokine induced by gamma interferon; MIP: macrophage inflammatory protein; RANTES: regulated on activation normal T cell expressed and secreted; SCF: stem cell factor; SDF: stromal cell-derived factor; TNF: tumor necrosis factor; EGF: epidermal growth factor; VEGF: vascular endothelial growth factor. IGF: insulin-like growth factor; IGFBP: insulin-like growth factor binding proteins; PDGF-BB: platelet-derived growth factor-BB; BDNF: brain-derived neurotrophic factor; BLC: B-lymphocyte chemoattractant; CK: chemokine; FGF: fibroblasts growth factors; Flt: fms-like tyrosine kinase; GCP: granulocyte chemotactic peptide; GDNF: glial cell line-derived neurotrophic factor; HGF: hepatocyte growth factor; IP: interferon-gamma-inducible protein; LIF: leukemia inhibitory factor; MIF: macrophage inhibition factor; NAP: neural antiproliferative protein; IL: interleukin; NT: neurotensin; PARC: pulmonary and activation-regulated chemokine; PIGF: placental growth factor; TARC: thymus and activation regulated chemokine; TGF: transforming growth factor; TIMP: tissue inhibitor of metalloproteinases.

**Figure 5:**
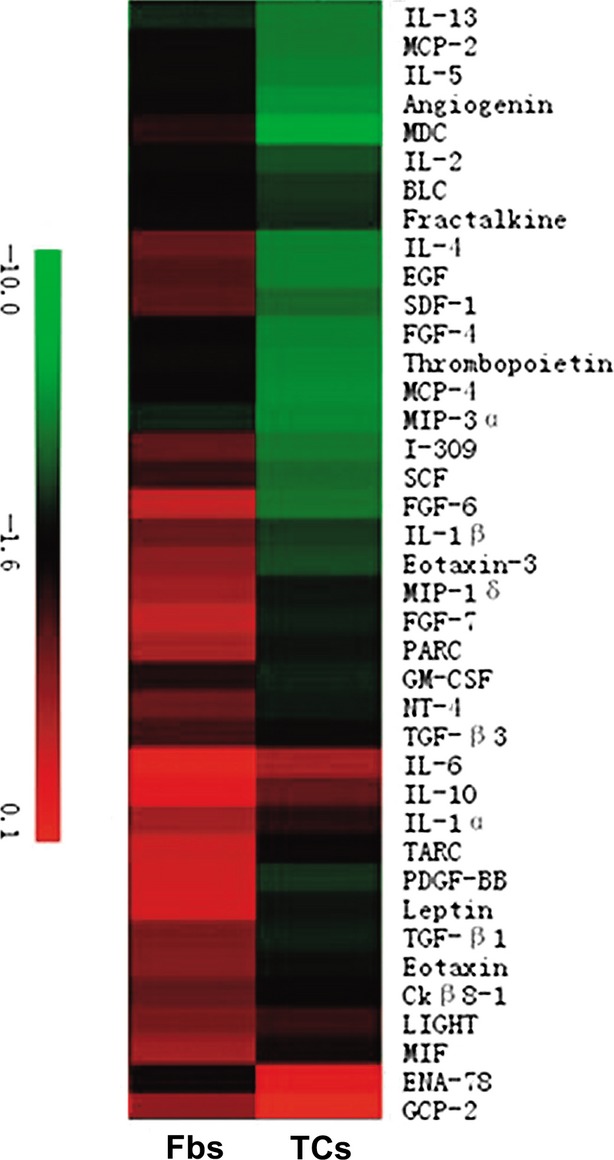
Hierarchical cluster analysis of the differentially expressed cytokines among telocytes (TCs) and fibroblasts (Fbs). The different colour represents relative expressions of different cytokines.

The supernatants of cultivated human dermal TCs and Fbs were analysed for cytokine profiling. Each array membrane contained 79 cytokines, as it is shown in Figure 4. The relative levels of cytokines expression were compared between TCs and Fbs. Only the levels that increased at least 1.5 times were considered significant for our study. Considering this, we found that 37 cytokines are highly expressed (more than 1.5 times) in Fbs than in TCs (Table1). Moreover, 6 of those 37 cytokines were found 9.5 times more expressed in Fbs than in TCs: *e.g*. interleukin 5 (IL-5), monocyte chemotactic protein-3 (MCP-3), MCP-4, macrophage inflammatory protein-3 (MIP-3a), angiogenin, thrombopoietin (Table1). On the other hand, two cytokines were expressed 1.5 times higher in TCs than in Fbs: neutrophil-activating peptide 78 (ENA-78) and granulocyte chemotactic protein-2 (GCP-2) (Table2). All 39 (37 + 2) cytokines differently expressed between TCs and Fbs were showed in the hierarchical cluster (Fig. 5).

**Table 1:** Cytokines up-regulated in human dermal Fbs in comparison with TCs

Cytokines	Fbs/TCs	*P*-value (Fbs/TCs)	Cytokines	Fbs/TCs	*P*-value (Fbs/TCs)
GM-CSF	2.990494572	0.004068158	Angiogenin	9.583094698	0.045069501
I-309	3.1226639	0.0209053	EGF	9.1678442	0.034149
IL-1β	4.2659416	0.0076248	PDGF-BB	2.5881332	0.0108735
IL-2	4.1940349	0.0162552	Leptin	2.5331073	0.006458
IL-4	7.4433261	0.0187587	BLC	3.7833313	0.025467
IL-5	13.319194	0.0462483	Ckβ8-1	2.2117157	0.0074857
IL-6	2.0135764	0.0042913	Eotaxin	2.0181175	0.031139
IL-7	2.3587741	0.0590858	Eotaxin-3	4.2737441	0.0168568
IL-10	1.7619581	0.003995	FGF-4	7.9130129	0.0129979
IL-13	6.5371889	0.0083464	FGF-6	4.5026618	0.0140899
MCP-2	7.3634195	0.0052858	FGF-7	4.9359943	0.0003372
MCP-3	9.9049627	0.0519322	Fractalkine	4.2430157	0.0216492
MCP-4	10.78187	0.0101099	MIP-1δ	3.6553571	0.001508
MDC	7.9091082	0.0027284	LIGHT	1.9522557	0.02218
SCF	3.8767312	0.0030059	MIF	2.0987014	0.0029001
SDF-1	8.1001679	0.0027454	MIP-3α	17.086088	0.0274467
TGF-β1	2.3181673	0.0113161	NT-4	3.4582038	0.0069577
TGF-β3	2.3968453	0.0193089	PARC	4.2626479	0.0016813
Thrombopoietin	11.187534	0.0069316			

Fbs: fibroblasts; TCs: telocytes; I-309: eosinophil chemo-attractant protein CCL-1; IL-1β: interleukin-1β; MCP-2: monocyte chemotactic protein-2; MDC: macrophage-derived chemokine; SCF: stem cell factor; SDF: stromal cell-derived factor; TGF: transforming growth factor; EGF: epidermal growth factor; PDGF-BB: platelet derived growth factor-BB; BLC: B-lymphocyte chemo-attractant; FGF-4: fibroblasts growth factor-4; MIP: macrophage inflammatory protein; MIF: macrophage inhibition factor; NT-4: neurotensin-4; LIGHT: tumor necrosis factor superfamily member 14 (TNFSF14), or CD258 in cluster of differentiation terminology. PARC: pulmonary and activation-regulated chemokine; GM-CSF: granulocyte-macrophage colony stimulating factor.

**Table 2:** Cytokines up-regulated in human dermal TCs in comparison with Fbs

Cytokines	TCs/Fbs	*P*-value (TCs/Fbs)	Cytokines	TC/Fib	*P*-value (TCs/Fbs)
ENA-78	2.1171218	0.0128784	GCP-2	1.7086424	0.0018594

TCs: telocytes; Fbs: fibroblasts; GCP-2: granulocyte chemotactic protein-2; ENA-78: epithelial-derived neutrophil-activating peptide 78.

ELISA analysis was performed to confirm that the levels of those six cytokines were 9.5 times overexpressed in Fbs in comparison with TCs (data not shown).

## Discussion

A large body of data recorded the presence of Fbs in stroma of various organs. Also, in other organs, the distinctiveness of TCs from different interstitial cells was documented concerning their aspect in cell culture 12,23, (ultra)structural features 20,36–38, microRNA profile 39, gene description 26,27,40, proteomics 25 and secretomics 41. In human skin, the presence of dermal TCs (papillary and reticular) was previously documented using various techniques 1,2,27–29. By TEM, in human dermis, TCs were constantly found in spatial relations with vascular structures, immune cells and/or adnexal structures 1,29.

In this study, by TEM and SEM, we documented the distinctiveness of TCs population in human dermis, based on their ultrastructural features and also their peculiar cytokine profile.

The ultrastructural characteristics, and also the differences between human dermal TCs and Fbs are in accordance with the previous ones reported in other organs. We found that TCs have a small cell body with Tps – the most particular ultrastructural features of TCs. Tps are very long (the measured average length on TEM images is of several hundreds of micrometres) and very thin and slender cellular prolongations of unequal calibre, with string of beads appearance. Adversely, Fbs are ultrastructurally featured by a large cell body of various aspects, with short and conical thick cellular prolongations.

Furthermore, the individuality of human dermal TCs and their distinctiveness than Fbs was proven using cytokine antibody array. Thirty-nine cytokines (out of 79 detected cytokines) were found to be differentially expressed between TCs and Fbs. These were at least 1.5 times overexpressed either in TCs or Fbs (37 in Fbs and 2 in TCs). Thus, we showed that the expression of these cytokines in TCs is dissimilar to that of Fbs.

The expression of ENA-78 and GCP-2 was found to be significantly higher in TCs than in Fbs. ENA-78 (epithelial neutrophil-activating peptide 78 or CXCL5) is a small neutrophil chemo-attractant cytokine being normally produced as a result of inflammatory stimulation. ENA-78 was also reported to have potential in regulation of angiogenesis 42,43. This finding is consistent with the previous data regarding TCs involvement in (neo)angiogenesis in heart 44–47. Moreover, the paracrine involvement of TCs in angiogenesis and/or vascular homoeostasis is also supported by the ultrastructural close proximity of their Tps with *ab luminal* faces of endothelial cells. Furthermore, TCs' positive expression for ENA-78 and also the TEM data showing TCs' close spatiality with mononuclear cells could represent the substrate for the attributed role of TCs in the integration of the intercellular signalling and immune modulation response. However, ENA-78 was credited with contribution in cancer cell proliferation, migration and invasion 48. Thus, it is tempting to believe the involvement of TCs in dermal tissue remodelling/repair processes. Nevertheless, such functional implications were previously hypothesized for TCs in skin and in other organs 3,9,10,13,29,44,45,49–55. Previous published data showed that the distribution and density of dermal TCs are affected in skin pathological conditions 27–29. Moreover, the normalization of their number is correlated with the remission of the disease. These data are supportive for the participation of TCs in reparatory processes of dermal tissue 29.

Granulocyte chemotactic protein 2 (or CXCL6) is another highly expressed cytokine by TCs. This small cytokine has documented function in granulocytes chemo-attraction 56. This confirms one more role of TCs in immune modulation of skin diseases. However, previous published data showed that functional inhibition of GCP-2 restrict melanoma growth and its metastases 57. Moreover, angiogenic properties of GCP-2 were previously described in heart, the level of GCP-2 being correlated with enhanced angiogenesis and reduction of infarct size 58. These data are in accordance with TEM data, positive expression of TCs for VEGF, NOS2, PDGFRα, or secretion of angiogenic miRNAs 1,29,47.

Adversely, 37 cytokines were highly expressed in dermal Fbs than in TCs. These cytokines elicit functions in chemotaxis for monocytes (MCP-3, MCP-4) 59–61, eosinophils (IL-5, MCP-4) 61, basophils (MCP-4) 62 and lymphocytes (MCP-4, MIP-3a) 61–63. Also, they are regulating the macrophages function (MCP-3) 59, or are involved in regulation of differentiation of megakaryocytes and platelets (trombopoietin) 64 or modulation of angiogenesis (angiogenin) 65. In conclusion, these cytokines are predetermined for either for the homoeostasis of interstitium or for regulation of first stages of wound healing. However, the cytokine profile we identified in this analysis is in accordance with the structural roles attributed to Fbs in connective tissue.

The cytokine profiles we identified in the present work for skin TCs and Fbs were different from the results got in the protein profiles comparison between human lung or cardiac TCs and Fbs 25,41. This is suggestive for the idea of cytokine expression specificity of TCs in relation with tissue type, and also for the existence of TCs subtypes 17. Here, we only detected 79 cytokines. Although they could cover most of the cytokines in the present time, this was only a limitation for the comparison of cytokine profiles between TCs and Fbs. However, in the set of cytokines we tested, we remarked the TCs susceptibility for higher secretion of CXC chemokines (*e.g*. CXCL 5 – ENA-78 and CXCL-6 – GCP-2) in comparison with Fbs, which have a higher secretion of CC chemokines (*e.g*. CCL-7 – MCP-3, CCL-13 – MCP-4, CCL-20 – MIP-3a).

We may conclude that considering their ultrastructural differences and divergent cytokine profiles, TCs and Fbs are different types of interstitial cells. The peaks of the cytokine expressions of TCs indicate, in skin, their functional roles as regulators of (neo)angiogenesis, and/or immune modulators, probably with implications in skin cancer progression. On the contrary, taking into account their cytokine profile, Fbs denote structural roles and also degrees of involvement in immune response. Our results are preliminary and could constitute a starting point for further studies. Of course, a more diverse spectrum of analyses, also including more cytokines species, would deepen the schism between these two different interstitial cells.
